# Does involving male partners in antenatal care improve healthcare utilisation? Systematic review and meta-analysis of the published literature from low- and middle-income countries

**DOI:** 10.1093/inthealth/ihz073

**Published:** 2019-10-15

**Authors:** Dedih Suandi, Pauline Williams, Sohinee Bhattacharya

**Affiliations:** Centre for Global Development and Institute of Applied Health Sciences, University of Aberdeen, Aberdeen AB25 2Z, UK; Indonesia Endowment Fund for Education (LPDP), Indonesia Ministry of Finance, Jakarta 10330, Indonesia; Centre of Academic Primary Care, University of Aberdeen, Aberdeen AB25 2ZL, UK; Centre for Global Development and Institute of Applied Health Sciences, University of Aberdeen, Aberdeen AB25 2Z, UK

**Keywords:** antenatal care, developing countries, male partner involvement, maternal health, newborn health, women’s health

## Abstract

**Background:**

Although in most low- and middle-income countries (LMICs) men are decision makers and control the household budget, their involvement in maternity care is limited. Reports from high-income countries indicate a beneficial effect of involving men in antenatal and delivery care on birth outcomes.

**Methods:**

We conducted a systematic review to assess whether similar effects are observed in LMICs. We searched MEDLINE, PubMed, CINAHL, Embase, NCBI, PsycInfo and other relevant databases using a comprehensive search strategy to retrieve relevant articles. A total of 17 articles were included. Meta-analysis of extracted data was performed, using the generic inverse variance method where possible. All studies were conducted in South Asia and Africa.

**Results:**

We found that involving a male partner in antenatal care was associated with skilled birth attendance utilization (pooled OR 3.19 [95% CI 1.55 to 6.55]), having institutional delivery (OR 2.76 [95% CI 1.70 to 4.50]) and post-partum visit uptake (OR 2.13 [95% CI 1.45 to 3.13]). Mother’s knowledge of danger signs and modern contraception utilization were also positively affected. However, it had no significant impact on the number of antenatal visits.

**Conclusions:**

Male involvement in antenatal care had a positive impact on the uptake of maternal health services. Further research needs to investigate whether this translates into improved maternal and newborn health in developing countries.

## Introduction

Maternal and newborn health are still major concerns worldwide. Around 99% of maternal deaths occur in low- and middle-income countries (LMICs).^1^ Complications during pregnancy and childbirth that contribute to maternal and infant mortality are preventable in many cases through appropriate care before and throughout pregnancy and delivery. The three delays model has been proposed for analysing the root cause of maternal mortality—delays in the decision to seek care, to reach care and to obtain care.^2^ Of these, the first delay in the decision to seek care sits squarely in the community, specifically on the person responsible for making household decisions.

In most developing countries, societies are patriarchal, with men having the role of primary decision maker and controlling the household budget. Thus the decision to seek care (the first delay) is usually made by the male partner or husband.^3^ Yet in some cultures, male members are not expected to be directly involved in their wife’s pregnancy and delivery care. If they are, this is considered by their peers as a demonstration of weakness.^4^ From a social perspective, the notion of joining one’s wife at the antenatal clinic is unusual in many communities and the husband’s presence is often considered superfluous.^5^ Vermeulen et al.^6^ reported that perceived traditional gender roles and a lack of knowledge and opportunities for involvement in obstetric care were some of the barriers to male partner involvement in rural Tanzania and can probably be generalized to other LMICs.

Recently, sexual and reproductive healthcare has moved from the age-old tradition of being woman-centric to being couple-centric. Two systematic reviews addressing the impact of male partner involvement on outcomes of pregnancy were published in 2015.^7,8^ Including seven primary studies in their systematic review, Aguiar and Jennings^7^ found a positive association between male partner attendance at antenatal clinic visits and women’s knowledge of danger signs during pregnancy, but it did not affect birth preparedness or utilization of antenatal care (ANC). The review by Yargawa and Leonardi-Bee^8^ found a significant reduction in post-partum depression and increased utilization of delivery and postnatal unhyphenated throughout care with male partner involvement. However, their review found that male presence in the delivery room was not associated with increased spontaneous labour and delivery. Based on these two systematic reviews, the World Health Organization strongly recommends male partner involvement in pregnancy and delivery to facilitate and support the care of women during pregnancy and delivery, accessing skilled birth attendance (SBA) and the timely use of facility care for obstetric and newborn complications. The idea of involving male partners in maternal and child health is designed as a means to support women’s access to care, address gender inequality and promote men’s positive involvement as partners and fathers.^9^ To date, interventions using mass media campaigns, counselling and outreach programmes involving both men and women in the community and workplace have met with modest success in improving birth outcomes, promoting gender equality and positively involving men in women and children’s health. This review focuses on the impact of involving male partners during ANC on the utilization of healthcare facilities, including SBA, at the time of delivery and in the postnatal unhyphenated throughout period.

## Methods

### Inclusion and exclusion criteria

First, a focused review question in the PECO (Participants, Exposure, Comparison, Outcome) format was formulated—‘What are the effects of male partner involvement in ANC on utilization of delivery and postnatal unhyphenated throughout care in low and middle income countries?’ Inclusion criteria were primary studies involving pregnant women and/or their partners in LMICs (according to the World Bank classification). A United Nation’s report defined male involvement in maternal and child health (MCH) as a social and behavioural change process that is required for men to play more responsible roles in MCH with the purpose of ensuring women’s and children’s well-being.^10^ In this review, male involvement in ANC is defined as the male partner’s/husband’s participation in ANC by escorting their girlfriends/wives and receiving any information regarding their pregnancy and health education during ANC. We hypothesized that male partners’ involvement in ANC will improve men’s awareness and increase their participation in all aspects of maternity and newborn care. Aborigo et al.^5^ argued that involving male partners in ANC allows men to have access to critical information on the reproductive health of their partners and on birth preparedness and could also increase adherence to guidance given at the clinic.

All quantitative study designs, including cross-sectional, case–control, cohort and randomised control trials, were eligible for inclusion. Those studies that were done in high-income countries, not reporting the effect of male involvement in ANC on the uptake of delivery and postnatal unhyphenated throughout care or solely focusing on the uptake of human immunodeficiency virus (HIV) testing/counselling were excluded.

### Search strategy

MEDLINE, PubMed, Scopus, CINAHL, Embase, NCBI, PsycInfo and Cochrane Collaboration—all mainstream electronic databases—were searched and used to retrieve the articles. No time or language limits were used. Search terms used the following keywords and MeSH headings: ‘male involvement/male participation’, ‘husband involvement’, ‘antenatal care’, ‘perinatal care’, ‘maternal health service’, ‘labour’, ‘delivery room’, ‘childbirth’, ‘new-born health’, ‘infant health’ and ‘developing countries’. Grouped terms such as ‘male involvement and ANC’ were also used. Boolean operators AND and OR were utilized as appropriate to connect the search terms.

Articles retrieved from electronic databases were screened for relevance based on their titles and abstracts as an initial step. Full texts were retrieved for the remaining articles and screened based on inclusion and exclusion criteria. Included references were imported into the reference management software RefWorks (ProQuest, Ann Arbor, MI, USA) and duplicates removed. Data from all included articles were extracted using a simple data extraction form. The extracted information consisted of author, year of publication, study setting, study aim, study design, study population, type of exposure or intervention, the number of participants, study outcomes and results of the study. The Downs and Black checklist for assessing study quality was used, as the primary studies included in the review varied by study design. The search strategy was developed and run independently by two reviewers (DS and PW) with the help of a medical librarian and were initially screened for relevance. Two reviewers (DS and PW or SB) independently assessed quality and extracted data from the included studies. Any disagreements were settled through discussion or by arbitration of the third reviewer. Meta-analysis of extracted data was done where appropriate using RevMan version 5.3 software (Cochrane, London, UK), and pooled ORs with 95% CIs were calculated for the outcomes of institutional delivery, SBA and postnatal unhyphenated throughout visit. ORs for male partner attendance at antenatal clinics were extracted from the primary studies where available and these were pooled using the generic inverse variance method. We assessed between-study heterogeneity using the *I*^2^ statistic and used a random effects model where heterogeneity was high, as evidenced by *I*^2^>50%. The search was rerun in May 2019 using the same search strategy and exclusion methods, and four new relevant papers were found.

## Results

### Search flow

**Figure 1:**
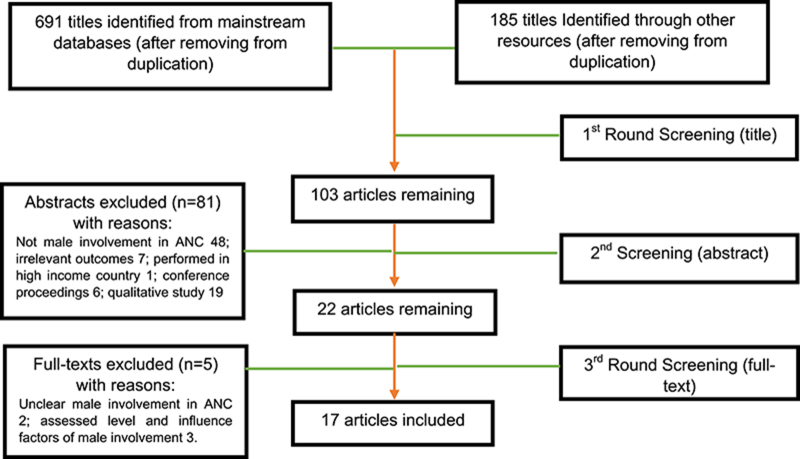
Article search flow.

After removing duplicates, 876 citations were identified from the electronic bibliographic database search and 103 articles remained after the initial screening of titles and abstracts. After the second round of screening, full texts of 22 potentially relevant articles were retrieved. In the final round, five articles were excluded. Two investigated the relationship between maternal mortality, birth preparedness/complication readiness and male involvement, but it was not clear whether male involvement included ANC attendance or not. Two articles investigated the level of male involvement and risk factors that contribute to male involvement. Another paper solely presented the influencing factors of male involvement in ANC. In the end, 17 articles were included. Details of the search flow are shown in Figure 1.

Table 1 presents the characteristics of the included studies. All included studies were conducted in Southeast Asia (n=8) or sub-Saharan Africa (n=9). Three studies were conducted in Nepal,^11–13^ two in India,^14,15^ one in Indonesia,^16^ one in Myanmar^17^ and one in Bangladesh,^18^ in the Southeast Asia region. In the Africa region, one study was conducted each in Kenya,^19^ Malawi,^20^ Zambia,^21^ South Africa,^22^ Burkina Faso^23^ and Uganda,^24^ while three studies were conducted in Ethiopia.^25–27^

**Table 1:** Characteristics of included studies

Reference	Country	Study design	Population	Intervention/exposure	Outcome	Effect measure
Chattopadhyay^14^	India	Cross-sectional	• Women 15–49 y of age (n=124 385)• Men (n=74 369) in India and three selected states	Husband’s pregnancy knowledge and wife’s participation in household decision making	Utilization of ANC and institutional delivery; women’s decision making in healthcare	Wives with husbands present during ANC had institutional delivery 1.35 times more than those without (OR 1.35, p<0.001)
Daniele et al.^23^	Burkina Faso	RCTs	Pregnant women 15–45 y of age, cohabiting with a man, gestational age 20–36 wk and expected to be able to give birth in a primary health centre,• intervention (n=583) and their partners• control (n=561)	Intervention in which the partner was invited:• interactive group discussion• couple’s counselling during pregnancy• postnatal unhyphenated throughout couple’s counselling sessions	Primary outcome:• attendance at postnatal unhyphenated throughout care consultations• exclusive breastfeeding• modern contraception usage	Intervention group was associated with higher rates of postnatal unhyphenated throughout care attendance (RR 1.23 [95% CI 1.11 to 1.37]), exclusive breastfeeding 3 mo post-partum (RR 1.35 [95% CI 1.15 to 1.59]) and effective modern contraception use 8 mo post-partum (RR 1.12 [95% CI 1.01 to 1.24])
Forbes et al.^25^	Ethiopia	Secondary data analysis	Women and men 15–49 y of age with a child 0–2 y of age:• women attended at least 1 ANC (n=1438)• men reported about ANC attendance (n=1204)	Male partner attendance at ANC	• Commenced ANC in the first trimester• attended at least 4 ANC appointments• received a urine test• received a blood test• were counselled about pregnancy complications• met these focused ANC guidelines	• There was no significant association between male attendance at ANC and commencing ANC in the first trimester. No male attendance was not significantly more likely to commence ANC in the first trimester than with male attendance (aOR 1.05 [95% CI 0.79 to 1.39])• There was no significant association between male attendance at ANC and receiving the recommended number of visits. No male attendance was not significantly more likely to have four or more ANC visits than with male attendance (aOR 1.06 [95% CI 0.82 to 1.38])• There was no significant association between male attendance at ANC and receiving all recommended components of ANC. No male attendance was not significantly less likely to receive all components of ANC than with male attendance (aOR 0.65 [95% CI 0.39 to 1.10])• Women attending ANC without a male partner were significantly less likely to report that they had been counselled about possible complications during pregnancy (aOR 0.64 [95% CI 0.48 to 0.86])• Women attending ANC without a male partner were significantly less likely to have a urine test than those with a male partner (aOR 0.73 [95% CI 0.55 to 0.97])• Women attending ANC without a male partner were significantly less likely to have a blood test than those with a male partner (aOR 0.70 [95% CI 0.53 to 0.93])
Kalembo et al.^20^	Malawi	Cohort	HIV-positive women with a male partner (n=65) and without a male partner (n=411)	• ANC clinic attendance,• receiving counselling for HIV• disclosing HIV result	• Uptake of PMTCT• condom use• place of delivery• infant HIV status (infected, uninfected, dead)	Male partner involvement was significantly associated with condom use (aOR 5.6 [95% CI 2.3 to 13.5]), hospital delivery (aOR 25.9 [95% CI 10.6 to 63.6]) and completion of follow-up in the programme (aOR 16.8 [95% CI 8.5 to 33.4])
Kashitala et al.^21^	Zambia	Retrospective cohort	Pregnant women who accessed ANC service March 2012–December 2012 (n=2007). Groups: ANC visit couples (n=220), ANC visit without partner (n=1787)	Male involvement in ANC	Having institutional delivery/SBA, postnatal unhyphenated throughout care visit attendance	Male partner involvement was significantly associated with delivery at a healthcare facility (OR 1.53 [95% CI 1.15 to 2.04]); post-partum visit attendance (OR 1.58 [95% CI 1.2 to 2.1])
Kunene et al.^22^	South Africa	Cluster RCT	Women who attended the ANC clinics in the interventions clinic: intervention group (n=995), control group (n=1081). Attended with a male partner: intervention group (n=608), control group (n=558)	• Improving the existing ANC services• introducing strengthened counselling for pregnant women and their partners	• Willingness of men to accompany their partners during antenatal and post-partum care• the effect of the accompaniment on family planning knowledge and use, on sexually transmitted infection (STI) knowledge and prevention, couple communication and male involvement, recognition of pregnancy danger signs and mother and baby’s healthcare at 6 mo post-partum	No significant difference found between intervention and control group on the use of contraception at 6 mo post-partum, the level of assistance given by male partners, knowledge of danger signs, immunization and baby feeding practice and in-risk behaviour for STIs or in condom use with either regular or non-regular partners
Mangeni et al.^19^	Kenya	Secondary data analysis	Couples that reported a birth in the 3 years before the survey (2008/2009) (n=873)	Male involvement in maternal health	Utilization of SBA	Women whose husbands attended at least one ANC visit were more likely to have SBA than those whose husbands did not attend an ANC visit (aOR 2.17 [95% CI 1.14 to 4.11])
Mohammed et al.^26^	Ethiopia	Community-based cross-sectional study	210 couples who recently had a baby <6 mo old and lived in Addis Ababa at least a year	Male involvement in maternal health services:• initiated discussion with partner about ANC/PMTCT• requested partner be tested for HIV• took time to find out what occurred during the partner’s ANC visits• reminded partner about ANC follow-up• covered costs of partner’s ANC visit• accompanied partner to ANC clinic at least once• physically entered the ANC room with partner, counselled and tested for HIV with partner, overall male partner involvement scale score	Primary outcome: maternal healthcare services utilization:• a minimum of one ANC attendance during the last pregnancy• the first ANC appointment in the first 12 wk of pregnancy• four or more ANC visits throughout pregnancy• tested for HIV during pregnancy• SBA• delivered at a healthcare facility	• Took time to find out what occurred during partner’s ANC visits associated‐ significantly with one or more ANC visit (aOR 5.17 [95% CI 1.19 to 22.48])‐ significantly with first ANC visit within the first trimester (aOR 1.93 [95% CI 1.04 to 3.60])‐ not significantly with less likely to have four or more ANC visits (aOR 0.82 [95% CI 0.41 to 1.62])‐ not significantly with tested for HIV (aOR 2.15 [95% CI 0.91 to 5.07])‐ significantly with SBA (aOR 2.93 [95% CI 1.24 to 6.9])‐ not significantly with having institutional delivery (aOR 1.95 [95% CI 0.93 to 4.08])‐ not significantly with less likely to utilize all services (aOR 0.77 [95% CI 0.37 to 1.59])• Accompanied partner to ANC clinic at least once associated‐ significantly with one or more ANC visit (aOR 5.49 [95% CI 1.07 to 28.20])‐ not significantly with first ANC visit within the first trimester (aOR 1.78 [95% CI 0.98 to 3.22])‐ not significantly with less likely to have four or more ANC visits (aOR 1.63 [95% CI 0.83 to 3.18])‐ significantly with tested for HIV (aOR 2.95 [95% CI 1.25 to 7.00])‐ not significantly with SBA (aOR 1.85 [95% CI 0.80 to 4.26])‐ not significantly with having institutional delivery (aOR 1.15 [95% CI 0.57 to 2.34])‐ not significantly with utilized all services (aOR 1.68 [95% CI 0.80 to 3.52])
						• Physically entered the ANC room with partner associated‐ not significantly with having one or more ANC visit (aOR 0.94 [95% CI 0.10 to 8.98])‐ not significantly with first ANC visit within the first trimester (aOR 1.01 [95% CI 0.49 to 2.44])‐ not significantly with four or more ANC visits (aOR 1.29 [95% CI 0.49 to 3.36])‐ not significantly with tested for HIV (aOR 1.81 [95% CI 0.35 to 9.23])‐ not significantly with SBA (aOR 1.20 [95% CI 0.81 to 1.77])‐ not significantly with having institutional delivery (aOR 7.58 [95% CI 0.92 to 62.20])‐ not significantly with utilized all services (aOR 1.02 [95% CI 0.36 to 2.86])• Overall male partner involvement scale score‐ associated significantly with having one or more ANC visit (aOR 1.61 [95% CI 1.05 to 2.45])‐ significantly with first ANC visit within the first trimester (aOR 1.19 [95% CI 1.03 to 1.39])‐ not significantly with less likely to have four or more ANC visits (aOR 0.98 [95% CI 0.83 to 1.15])‐ significantly with tested for HIV (aOR 1.52 [95% CI 1.18 to 1.96])‐ significantly with SBA (aOR 1.44 [95% CI 1.13 to 1.84])‐ significantly with having institutional delivery (aOR 1.22 [95% CI 1.01 to 1.48])‐ not significantly with less likely to utilize all services (aOR 0.97 [95% CI 0.82 to 1.15])
Mullany et al.^11^	Nepal	RCT	Pregnant women with gestational age 16–28 wk attending their first ANC visit whose husbands were present at the hospital compound (n=442). They should be ≥18 y and live <90 min from the hospital. Group A: couples (n=145), group B: women alone (n=148), group C: control (n=149)	Male involvement in antenatal health education	• Birth preparedness (purchased safe delivery, save money for delivery etc.)• utilization of SBA• number of ANC visits• post-partum visit attendance	Women in the couples group were more likely to be highly prepared for birth control than those in the control group (RR 5.19 [95% CI 1.86 to 14.53]). Women in the couples group were 1.29 times more likely to attend a post-partum visit than women in the control group (RR 1.29 [95% CI 1.04 to 1.60]). However, the impact of the intervention was not statistically significant for utilization of SBA, number of ANC visits and attending SBA
Mullany et al.^12^	Nepal	RCT	Women presenting at hospital from August 2003 to January 2004. Group A: couples (n=145), group B: women alone (n=148), group C: control (n=149)	• Antenatal health education	Maternal and reproductive health knowledge level	Women educated with husband had more knowledge of pregnancy complications at the time of follow-up compared with the women-alone group (RR 1.15 [95% CI 1.0 to 1.32]), were slightly more likely to have high knowledge scores at follow-up compared with women educated alone (RR 1.18 [95% CI 1.01 to 1.38]), were more likely to have improvement in knowledge from baseline to follow-up than those who were in the control group (RR 1.25 [95% 1.04 to 1.51])
Rahman et al.^18^	Bangladesh	Cross-sectional	Married women 19–49 y of age with a birth history in the 12-mo period preceding the survey, recruited from another larger study, as this study was embedded	Male accompanying in• ANC• during childbirth• postnatal unhyphenated throughout• care and care seeking for complications during pregnancy	• Level of knowledge of women and their husbands regarding MNH issues• engagement of women and their husband• level of involvement of husbands in terms of accompanying their wives• association between husband’s knowledge and accompanying their wives while receiving maternal and neonatal health (MNH) services• association between their involvement and women’s utilization of skilled MNH services	• Women who received ANC with their husband were more likely to get ANC from a medically trained provider (OR 4.7 [95% CI 2.5 to 9.0], aOR 4.5 [95% CI 2.3 to 8.7])• Women who were accompanied by their husband during childbirth had an OR 2 times higher in having delivery at a health facility than those without their husband (OR 2.0 [95% CI 1.1 to 3.9], aOR 1.5 [95% CI 0.8 to 3.1])
Sapkota et al.^13^	Nepal	Non-randomized controlled trial	Women (primigravida) admitted to the hospital. Mixed support group (n=11), couples group (n=97), with female friend group (n=96), control group (not accompanied; n=105)	Accompaniment during childbirth	Feeling in control during labour, Labour Agentry Scale (LAS)	The husband’s presence during childbirth was more positively related to the women’s feeling of being control (β=0.57, p<0.001). Also, those who gave birth with their husband’s support reported higher mean LAS scores (47.92±6.95) than those with female friend’s support and the woman in the control group
Teklesilasie and Deressa^27^	Ethiopia	Prospective cohort	709 antenatal women in the beginning with gestational age between 24 and 36 wk, living with their husband at least a year. At the end of follow-up, 664 women were included for analysis. Intervention group (n=385), control group (n=279)	Husband’s involvement in ANC	SBA utilization	Women accompanied by their husbands to ANC were 6.27 times more likely to use SBAs during birth (OR 6.33 [95% CI 4.5 to 8.9], aOR 6.27 [95% CI 4.2 to 9.3])
Timsa et al.^24^	Uganda	Cross-sectional	All women who had delivered a child in the past 12 mo and were living in one of the selected villages: Iganga (n=394), Luuka (n=797) or Buyenda (n=819)	Strategies for helping families prepare for birth: presence in delivery room	Determinants of birth preparedness	Being prepared for birth was associated with having four ANC visits (aOR 1.92 [95% CI 1.1 to 1.83]), attendance at ANC during the first (aOR 1.94 [95% CI 1.09 to 3.44]) or second trimester (aOR 1.87 [95% CI 1.09 to 3.22]), counselling on danger signs (aOR 2.07 [95% CI 1.57 to 2.74]), accompanied by husband to place of delivery (aOR 1.47 [95% CI 1.15 to 1.89]), higher economic status (aOR 2.04 [95% CI 1.38 to 3.01]) and having a regular income (aOR 1.83 [95% CI 1.2 to 2.79])
Wai et al.^17^	Myanmar	Cross-sectional	Husbands ≥18 y of age who had at least one child within 2 years at the time of the interview	Male involvement in ANC attendance, financial support, birth preparedness, accompaniment to the delivery room and postnatal unhyphenated throughout care accompaniment	Maternal healthcare services utilization: having ANC more than four times, having institutional delivery/SBA and receiving postnatal unhyphenated throughout care	Increased utilization of maternal healthcare services was found among women who were accompanied by husbands to ANC (aOR 5.82 [95% CI 3.34 to 10.15]) and those who had a birth plan (aOR 2.42 [95% CI 1.52 to 5.47])
Wicaksono^16^	Indonesia	Secondary data analysis	Women 15–49 y of age who had their last childbirth in the year prior to the survey (n=4000)	Husband participation in ANC	Utilization of SBA	Women whose husbands attended one or more ANC visit were more than 2 times likely to use SBA than those whose husbands did not (aOR 2.186 [95% CI 1.786 to 2.702])
Varkey et al.^15^	India	RCT	Pregnant women who attended ANC in the selected clinic sites. Intervention group (n=581), control group (n=486)	• ANC counselling• STI preventive counselling• universal syphilis testing• provision of STI services and treatment• post-partum care and family planning counselling	• The willingness of men to escort their partner to a programme during antenatal and post-partum care• the effect of the accompaniment on family planning knowledge and use, on STI knowledge and prevention, and on child and maternal health indicators	Women in the intervention group had a higher level of knowledge of obstetric danger signs than women in the control group (z=3.02, p<0.05). No significant difference in the proportion of infants immunized in both groups and the percentage of women exclusively breastfeeding until 6 mo was significantly less in the intervention group than in the control group (Zw=−2.60, p<0.05)

STI, sexually transmitted infection.

In terms of study design, there were five randomized controlled trials (RCTs),^11,12,15,23^ of which one was a cluster RCT,^22^ one was a non-randomized controlled trial^13^ and the others were all cohort studies or cross-sectional surveys.

In all the included studies, the population was women of reproductive age, although some were recruited from antenatal clinics while others participated in surveys after pregnancy and delivery. All the studies compared couple attendance at ANC with attendance by the pregnant woman alone. The outcomes reported included institutional delivery,^11,14,17,20,21,26^ SBA,^11,16,17,19,27^ and post-partum visit.^11,17,21,23^ Other outcomes reported included birth preparedness, knowledge about maternal and reproductive health, newborn immunization, exclusive breastfeeding and contraceptive use post-partum. Kalembo et al.,^20^ in their study including HIV-positive women, also reported on the uptake of prevention of mother-to-child transmission (PMTCT) and HIV status of the newborn.

The Downs and Black checklist for assessing study quality was used, as the primary studies included in the review varied by study design. The majority of papers scored ≥13 out of 26. The ones that scored <13 were Chattopadhyay^14^ (10), Forbes et al.^25^ (10), Mohammed et al.^26^ (8), Timsa et al.^24^ (8), Wai et al.^17^ (10) and Wicaksono^16^ (12). The average score of the studies was 14/26; the highest scoring study was Daniele et al.^23^ (23) and the lowest scoring studies were by Timsa et al.^24^ and Mohammed et al.^26^ (8). The external validity criteria scored proportionately lower (22%) compared with other criteria in the checklist and as a percentage of the total possible points collected (49%).

### Maternal healthcare utilization

Male partner involvement in pregnancy care appeared to have the greatest effect on healthcare utilization.

#### Number of ANC visits

In terms of the number of ANC visits, Mullany et al.^11^ showed that there was no significant impact of male involvement. However, in Myanmar, women who were accompanied by their partner to an ANC visit were more likely to have more than four ANC visits during pregnancy (adjusted OR [aOR] 5.82 [95% CI 3.34 to 10.15]).^17^ In Ethiopia, the absence of male partners in ANC was not significantly associated with ANC commencement in the first trimester (aOR 1.05 [95% CI 0.79 to 1.39]) or having four or more ANC visits (aOR 1.06 [95% CI 0.82 to 1.38]).^25^ Similarly, the absence of male partners during ANC was not significantly linked to less likelihood of receiving all components of ANC (aOR 0.65 [95% CI 0.39 to 1.10]).^25^ A study in Bangladesh demonstrated that women accompanied by their husband were 4.5 times more likely to have ANC from a medically trained provider (OR 4.7 [95% CI 2.5–9.0]; aOR 4.5 [95% CI 2.3 to 8.7]).^18^

#### Institutional delivery

**Figure 2:**
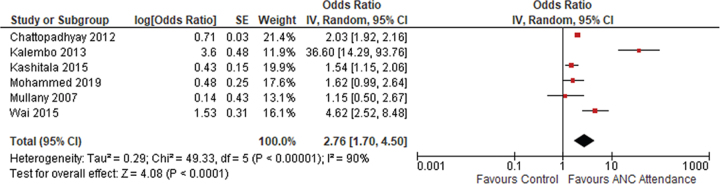
Meta-analysis of having institutional delivery.

The pooled OR (2.76 [95% CI 1.70 to 4.50]) from five included studies conducted in separate countries in Southeast Asia and Africa was statistically significant. However, the differences between the effect of the individual study and the pooled effect across studies were considerable (*I*^2^=90%). Therefore the random effects model was applied. Figure 2 shows the effect of male partner involvement in ANC with the chance of having an institutional delivery.

#### SBA

**Figure 3:**
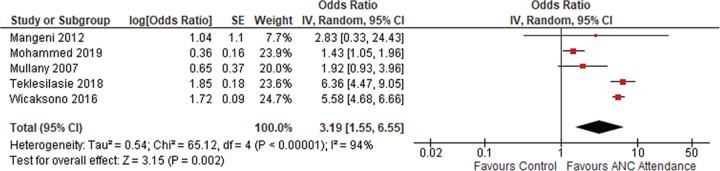
Meta-analysis of utilization of SBA.

Similar to institutional delivery, the pooled aOR for SBA was statistically significant (3.19 [95% CI 1.55 to 6.55]) using a random effects model, as *I*^2^ was 94% (Figure 3). Five studies showed a positive association between male ANC attendance and utilization of SBA. Kashitala et al.^21^ conducted a cohort study in Zambia and found that women with male accompaniment had higher odds than unaccompanied women (OR 1.53, p<0.005) of arranging for skilled attendance at delivery. This evidence was supported by other studies in Kenya that revealed women with a partner were more likely to have SBA (aOR 2.17 95% CI 1.14 to 4.11]).^19^ In India (aOR 1.35 [95% CI 1.14 to 4.11]),^14^ Ethiopia (aOR 6.27 [95% CI 4.2 to 9.3])^27^ and Indonesia (aOR 2.17 [95% CI 1.77 to 2.70]),^16^ similar patterns were observed. Kalembo et al.^20^ found that accompanied women had increased odds of having a hospital delivery than those who were unaccompanied (aOR 25.9, p<0.001). In contrast, Mullany et al.^11^ and Wai et al.^17^ found no significant differences between women with and without a partner.

**Figure 4:**
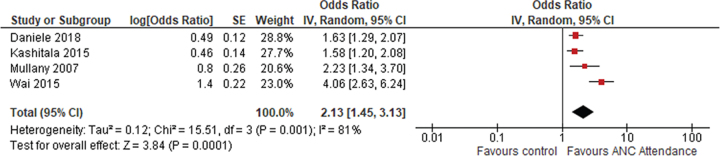
Meta-analysis of post-partum visit.

#### Post-partum visits

Involving male partners in ANC was associated with increasing post-partum care attendance. The pooled OR for postnatal unhyphenated throughout visits was found to be 2.13 (95% CI 1.45 to 3.13) using a random effects model, as heterogeneity was 81% (Figure 4). In Zambia, women who came with their spouse for ANC visits were 58% more likely to use post-partum care.^21^ Women who received antenatal health education along with their partners were more likely to attend a post-partum visit than those who received education alone (RR 1.25 [95% CI 1.01 to 1.54]) in Nepal,^11^ whereas a study in Myanmar found no significant impact of male partner involvement on receiving post-partum care.^17^

#### Effective modern contraception usage

Daniele et al.^23^ investigated the effect of male involvement during an antenatal education session on the utilization of effective modern contraception 8 mo post-partum. Inviting male partners into antenatal education sessions positively affected the use of effective modern contraception in women, with the effect size 1.12 greater than for women in the control group (RR 1.12 [95% CI 1.01 to 1.24]).

### Reproductive health knowledge and birth preparedness

#### Knowledge of miscarriage and danger signs

Four studies reported conflicting results regarding the effect of male involvement on knowledge of miscarriage and danger signs. In Nepal, Mullany et al.^12^ found that women who had antenatal education with their partner were likely to know more about pregnancy complications (aRR 1.15 [95% CI 1.00 to 1.32]) and were significantly more likely to show improvement in knowledge from baseline to follow-up (RR 1.25 [95% CI 1.04 to 1.51]) compared with those who attended alone. Similar results were found in India. Inviting a male partner into ANC counselling significantly increased the knowledge of pregnancy danger signs in 24% of women 13% of control women (z=2.99, p<0.05).^15^ In Ethiopia, women who attended ANC without a male partner were less likely to report that they had been counselled about possible pregnancy complications (aOR 0.64 [95% CI 0.48 to 0.86]).^25^ In contrast, in South Africa, Kunene et al.^22^ found no difference between couples who received and did not receive ANC counselling in knowledge of miscarriage or obstetric danger signs (p>0.05).

#### Birth preparedness

One study investigated the association between male involvement and birth preparedness (purchased safe delivery, save money for delivery, having blood donor, etc.).^11^ Women who were educated with a spouse were 1.3 times more likely to make more than three birth preparations as compared with those who received education alone, although the effect was not statistically significant (aRR 1.30 [95% CI 0.78 to 2.15]).^11^

### Newborn health outcome

Articles that were directly related to newborn outcomes were mostly conducted in HIV-positive pregnant women.

#### Breastfeeding initiation

In India, an intervention involving the male partner in ANC significantly increased breastfeeding initiation within the first hour after birth in 63.1% of women in the intervention arm vs 47.3% of women in the control arm (z=3.28, p<0.05).^15^ A recent study from Daniele et al.^23^ in Burkina Faso showed similar findings. Women in the intervention arm were 1.35 times more likely to breastfeed exclusively compared with those who only received routine care (RR 1.35 [95% CI 1.15 to 1.59]).^23^ In contrast, in South Africa there was no significant difference found in terms of breastfeeding initiation between those who came with or without a man (p=0.1).^22^

## Discussion

Not all studies included in this review showed that male involvement had a significant impact on maternal and child health. However, most studies reported that men’s participation in ANC is more likely to have a positive impact on improving healthcare utilization in terms of SBA, institutional delivery and post-partum care. The number of ANC visits and birth preparedness were not significantly influenced by male involvement. HIV survival and breastfeeding initiation are newborn outcomes that were affected positively by male partner involvement. While male involvement in ANC had no impact on infant mortality and vertical transmission of HIV infection, male presence during labour had a positive impact on relieving the stress of the mother during childbirth and resulted in a higher proportion of spontaneous labours.

This review found that knowledge improvement was greater among those who had a male partner accompanying them than those who did not. This finding was similar to evidence in Tanzania, where male involvement improved knowledge of danger signs and birth preparedness.^28^ The relationship between couples and the shared decision-making process may be responsible for the impact of male partner involvement seen in knowledge improvement.^29^ For this reason, women who were educated with their male partners were more likely to share information together and have a discussion with their partner regarding pregnancy and delivery. Furthermore, male accompaniment in health education may aid retention of knowledge and increase a couple’s communication.^7^ All these reasons help explain how men’s involvement is more likely to improve women’s knowledge.

Addressing gender inequality is one of the recognized strategies to improve maternal and child health^30^ and is supported by Sustainable Development Goal 5. This not only focuses on women’s empowerment, but also the support of men, since gender is a social construct that affects both men and women.^29^ ANC is the first step for raising awareness of both the mother and father about maternal and infant health. Men, as the chief decision makers in patriarchal societies, have the potential to prevent the first delay in the three-delays model proposed by Dudgeon and Inhorn.^31,32^

Men are dominant in the patriarchal household and responsible for the planning and provision of healthcare for household members. Antenatal classes could be a way to change men’s knowledge and views towards maternal and infant health. In this context, it would be helpful to define the role of the male partner in pregnancy care. It is obviously more than just accompanying the pregnant woman for antenatal visits, and implies joint decision making with regard to pregnancy and birth planning for the benefit of the mother and the newborn.

This review also found that the presence of male partners in the delivery room had a positive impact on women’s feeling of being in control during childbirth,^13^ and their accompaniment during childbirth was positively linked to having an institutional delivery.^18^ Several studies have shown that male involvement during labour shortens the labour and reduces the epidural rate.^33^ Studies have demonstrated that the presence of social support is a significant factor in women’s adjustment to the stress of childbearing.^34^ In addition, Nepalese women felt the presence of their husband gave them greater self-confidence and relief of stress, and facilitated communication.^35^ The husband’s love and emotional support were expected by women during pregnancy and delivery.^36^ In contrast, a study in Hong Kong found that husbands’ involvement during labour was associated with a higher dosage of analgesia.^34^

Men’s engagement in different reproductive programmes resulted in positive outcomes, such as increased condom use, more couples following the PMTCT HIV transmission programme and the use of SBA.^37^

A comprehensive and inclusive literature search based on a focused review question and inclusion and exclusion criteria agreed to a priori are major strengths of this review. On the other hand, restricting the publications to the English language in the interest of time and resource constraints may have resulted in some relevant articles being missed. Most of the included literature came from sub-Saharan Africa and South Asia and thus the findings may not be generalizable to other LMIC settings. Since involving the male partner in pregnancy and delivery care is an intervention, it should, in an ideal world, be evaluated by randomized controlled trials. However, many of the studies included in this review were observational studies, which are prone to bias and confounding. Few of these controlled for confounding factors and reported adjusted effect estimates. We also cannot rule out potential publication bias, as studies with negative findings are less likely to be published. The outcomes assessed in the primary studies included in the review were most often process outcomes, such as an increase in knowledge or birth preparedness. While some of these process outcomes, such as SBA, have been shown to be effective in reducing maternal and perinatal mortality, others have no such evidence base. It is difficult to firmly conclude whether male partner involvement in pregnancy and childbirth improves birth outcomes for both the mother and the baby, as it is entirely plausible that the women who are accompanied by their male partners are already equal partners in the decision-making process and it is this empowerment that bestows the beneficial effects, not the involvement of the partner *per se*.

## Conclusions

In developing countries, male accompaniment in ANC has a beneficial impact on improving the mother’s knowledge of danger signs, utilization of an SBA and relieving stress and anxiety during labour, as well as increasing uptake of post-partum care and initiation of breastfeeding. However, further research needs to unpack the psychosocial elements that underpin the impact of male partner involvement in maternity care.
